# Predictors of Rehabilitation Service Utilisation among Children with Cerebral Palsy (CP) in Low- and Middle-Income Countries (LMIC): Findings from the Global LMIC CP Register

**DOI:** 10.3390/brainsci11070848

**Published:** 2021-06-25

**Authors:** Mahmudul Hassan Al Imam, Israt Jahan, Mohammad Muhit, Denny Hardianto, Francis Laryea, Amir Banjara Chhetri, Hayley Smithers-Sheedy, Sarah McIntyre, Nadia Badawi, Gulam Khandaker

**Affiliations:** 1 CSF Global, Dhaka 1213, Bangladesh; physiomahmud@yahoo.com (M.H.A.I.); arda.jahan89@gmail.com (I.J.); mmuhit@hotmail.com (M.M.); 2 Asian Institute of Disability and Development (AIDD), University of South Asia, Dhaka 1213, Bangladesh; 3 School of Health, Medical and Applied Sciences, Central Queensland University, Rockhampton, QLD 4701, Australia; 4 Central Queensland Public Health Unit, Central Queensland Hospital and Health Service, Rockhampton, QLD 4700, Australia; 5 CSF Global Indonesia, Waikabubak 87214, Indonesia; dennydrgmph@gmail.com; 6 The Salvation Army Rehabilitation Centre, Begoro EF0007, Ghana; francislaryea.fl@gmail.com; 7 CSF Global-Nepal, Balaju, Kathmandu 44600, Nepal; amirbanjara@yahoo.com; 8 Discipline of Child and Adolescent Health, Sydney Medical School, The University of Sydney, Sydney, NSW 2145, Australia; hsmitherssheedy@cerebralpalsy.org.au (H.S.-S.); nadia.badawi@health.nsw.gov.au (N.B.); 9 Cerebral Palsy Alliance Research Institute, The University of Sydney, Sydney, NSW 2050, Australia; SMcIntyre@cerebralpalsy.org.au

**Keywords:** cerebral palsy (CP), children, rehabilitation, factors, global, low- and middle-income country (LMIC)

## Abstract

Background: We assessed the rehabilitation status and predictors of rehabilitation service utilisation among children with cerebral palsy (CP) in selected low- and middle-income countries (LMICs). Methods: Data from the Global LMIC CP Register (GLM-CPR), a multi-country register of children with CP aged <18 years in selected countries, were used. Descriptive and inferential statistics (e.g., adjusted odds ratios) were reported. Results: Between January 2015 and December 2019, 3441 children were registered from Bangladesh (*n* = 2852), Indonesia (*n* = 130), Nepal (*n* = 182), and Ghana (*n* = 277). The proportion of children who never received rehabilitation was 49.8% (*n* = 1411) in Bangladesh, 45.8% (*n* = 82) in Nepal, 66.2% (*n* = 86) in Indonesia, and 26.7% (*n* = 74) in Ghana. The mean (Standard Deviation) age of commencing rehabilitation services was relatively delayed in Nepal (3.9 (3.1) year). Lack of awareness was the most frequently reported reason for not receiving rehabilitation in all four countries. Common predictors of not receiving rehabilitation were older age at assessment (i.e., age of children at the time of the data collection), low parental education and family income, mild functional limitation, and associated impairments (i.e., hearing and/or intellectual impairments). Additionally, gender of the children significantly influenced rehabilitation service utilisation in Bangladesh. Conclusions: Child’s age, functional limitation and associated impairments, and parental education and economic status influenced the rehabilitation utilisation among children with CP in LMICs. Policymakers and service providers could use these findings to increase access to rehabilitation and improve equity in rehabilitation service utilisation for better functional outcome of children with CP.

## 1. Introduction

Cerebral palsy (CP) is a group of disorders of movement and posture caused by non-progressive lesions in the developing brain [1]. Globally, an estimated 50 million people have CP, and the burden of CP is substantially higher in low-and middle-income countries (LMICs) [2,3,4,5]. Children with CP and their families can benefit from working with a multidisciplinary team of rehabilitation professionals to tackle impairments, activity limitations, and participation restrictions [6]. However, this is problematic in LMICs, where there are severe shortages of rehabilitation professionals and services [7].

Despite the large burden, there is little information describing the rehabilitation needs of children with CP in LMICs [6,8]. The existing evidence on rehabilitation service utilisation are largely from high-income countries (HICs). Limited available data indicate that a large proportion of children with CP in LMICs do not have access to rehabilitation services [3,9]. Moreover, rehabilitation service availability and utilisation among children with CP vary substantially between LMICs [10,11]. However, where service exists, the rehabilitation services utilisation is influenced by sociodemographic (e.g., age, sex, parental education), economic (e.g., family income), and environmental (e.g., transportation system) factors [12]. Nevertheless, young age (i.e., early diagnosis), parental education, and financial status have been found positively associated with rehabilitation service utilisation among children with CP in both HICs [13,14,15,16] and LMICs [9,10].

Understanding the magnitude of rehabilitation needs and potential factors influencing service utilisation is essential for planning, policymaking, and developing a comprehensive rehabilitation program in order to address the future needs of children with disabilities in LMICs [17]. To meet this important knowledge gap, comparable evidence on the rehabilitation status of children with CP and predictors of rehabilitation service utilisation in different LMICs is essential. This study aimed to determine the access to rehabilitation services and predictors of rehabilitation service utilisation among children with CP in selected LMICs.

## 2. Materials and Methods

This study utilised data collected as part of the Global LMIC CP Register (GLM-CPR), an ongoing collaboration between CP registers based in LMICs. A protocol clearly outlining case definition, methodology, and definition of core variables has been used to allow harmonisation of country-specific CP register data to the GLM-CPR dataset. As part of the GLM-CPR, detailed information related to rehabilitation service receipt among participating children is also documented since different types of services with varied cost, scope, and access are available for children with disability (e.g., CP) in the respective countries. In this study, data from four countries (i.e., Bangladesh, Nepal, Indonesia, and Ghana) included in the GLM-CPR were analysed to report the rehabilitation status and predictors of utilisation of the available rehabilitation services among children with CP in LMICs.

### 2.1. Case Definition Used in the GLM-CPR

The GLCMPR follows the case definitions of the Surveillance of CP in Europe (SCPE) and the Australian CP Register (ACPR) for the inclusion of children with CP in respective registers [18,19]. Children aged <18 years, who meet the case definition, are included in the respective CP registers of the GLM-CPR [20].

### 2.2. Surveillance Mechanisms and Data Collection Technique

The GLMCPR employs both population-based surveillance and hospital/institution-based surveillance mechanisms to recruit children with CP from LMICs.

In the population-based surveillance mechanism, the key informant method (KIM) is being used to identify children with suspected CP from communities. The KIM is a validated method with a 77.6% case-ascertainment rate [21] and has been reported as an effective community-based recruitment strategy for children with CP in Bangladesh [3] and Indonesia [11]. The following LMIC CP registers have adopted the KIM.

The Bangladesh CP Register (BCPR), a population-based surveillance of children with CP, represents rural and semi-urban Bangladesh (where the majority of the population (76.7%) lives) in terms of demographic and other indicators (e.g., birth rate, immunisation rate, perinatal mortality rate, literacy rate). The surveillance site covers 18 sub-districts (~4663 square km area, estimated total population ~6,243,989 and child population aged <18 years ~2,597,365) of seven districts in Bangladesh. Children registered into the BCPR between January 2015 and December 2019 were included in this study.

In Indonesia, a community-based KIM survey was conducted in eight districts of Sumba Island (~1480·5 square km, estimated total population ~328,600 and child population aged <18 years ~152,471) in 2017. Children registered between March 2017 and August 2017 through the KIM survey were included in this study.

Established in June 2018, the Nepal CP Register (NCPR) is an ongoing surveillance of children with CP in Nepal. The surveillance site covers six municipalities of Gorkha district (~823 square kilometers, total population ~184,546 and child population aged <18 years ~83,047). Children registered between June 2018 and October 2018 were included in this study.

In the hospital/institution-based surveillance mechanism, children with CP are identified prospectively following a convenience/purposive recruitment strategy during regular service provision in health facilities/rehabilitation centres. This method is being used to register children with CP living in Begoro town of Fanteakwa district (~1066 square km, total population ~133,301 and child population aged <18 years ~62,450) in Ghana. Children recruited between October 2018 and May 2019 into the Ghana CP Register (GCPR) were included in this study. The details about the study settings of all four CP registers included in the GLM-CPR have been published elsewhere [20].

In both types of surveillance mechanisms, children with suspected CP identified by key informants in the community or by trained health care professionals in the hospital setting undergo a detailed neurodevelopmental assessment by a multi-disciplinary medical assessment team including a paediatrician, a physiotherapist, and a counsellor for a confirmed diagnosis.

### 2.3. Study Variables

Using a uniform template, data are collected on selected core variables by each CP register. Variables included in this study were: sociodemographic characteristics (i.e., age and gender of children, educational level of parents, and monthly family income) and clinical characteristics (i.e., Gross Motor Function Classification System (GMFCS) level, Manual Ability Classification System (MACS) level, predominant motor type, topography, and associated impairments). Information on rehabilitation (i.e., whether the child ever received rehabilitation, the type of service received, the primary service provider, age of commencement of rehabilitation, and reasons for not receiving rehabilitation) were also collected from primary caregivers. In this study, the main outcome variable was the rehabilitation status (i.e., whether s/he ever received rehabilitation) of a child with CP recruited in any of the registers. Responses were collected as a binary variable with either ‘yes’ or ‘no’. Children who received at least one rehabilitation session were categorised as rehabilitation recipients and marked ‘yes’. Additionally, available medical records were reviewed for any documentation on rehabilitation services. Regarding reasons for not receiving rehabilitation, if a primary caregiver mentioned that s/he was not aware of the need or benefit of rehabilitation/intervention for her/his child with CP, or the availability of services, it was documented as ‘lack of awareness’. If a respondent mentioned financial hardship/lack of money for not receiving rehabilitation services of their child, we documented financial constraint as a reason for not receiving rehabilitation services. Similarly, if caregivers responded that they could not take their children to the available rehabilitation service centres because of the difficulties in transport use, then it was documented as a transport problem.

### 2.4. Data Management and Statistical Analysis

Continuous variables were collected as exact values and later recoded and categorised into groups (e.g., age in years with two decimals was recoded into 0–4 years, 5–9 years, 10–14 years, and 15–18 years groups). Similarly, the monthly family income data were collected in local currencies and then converted to USD and categorised into three monthly family income groups (i.e., US$0–50, US$51–100, and US$ >100). Descriptive analyses were performed to report the overall rehabilitation status. Bivariate analysis was completed to identify the underlying factors of rehabilitation services. Factors that were found significant in unadjusted analyses were fitted into the adjusted model. Adjusted odds ratios (aOR) with 95% confidence interval (CI) were reported. A *p* value < 0.05 was considered significant. All data were analysed using Statistical Package for the Social Sciences (SPSS) software version 26 (IBM, Armonk, NY, USA).

### 2.5. Ethics

Ethical approval was obtained from the international/national/institutional Human Research Ethics Committee (HREC) for each of the countries registered with the GLM-CPR before the commencement of the registers. For the BCPR, ethical approval was taken from the Cerebral Palsy Alliance HREC (Reference no.: 2015-03-02) in Australia, the Asian Institute of Disability and Development (AIDD) HREC (Reference no.: southasia-irb-2014-l-01), and the Bangladesh Medical Research Council National Research Ethics Committee (BMRC/NREC/2013-2016/1267) in Bangladesh. For the NCPR, ethical approval was obtained from the Nepal Health Research Council Ethical Review Board (NHRC ERB) (Registration no.: 101/2018). In Indonesia, ethical approval was taken from the AIDD HREC (Reference no.: sothasia-irb-2017-1-01) and Hasanuddin University HREC, Indonesia (Reference no. 630/H4.8.4.5.31/PP36-KOMETIK/2017). For the GCPR, ethical approval was obtained from the Office of the Administrator Begoro Rehabilitation Centre. Prior to data collection, informed written consent was taken from primary caregivers of children with CP.

## 3. Results

This study included 3441 children with CP (2852 from Bangladesh, 182 from Nepal, 130 from Indonesia, and 277 from Ghana). The mean age at assessment was 7 years (y) and 9 months (mo) (standard deviation (SD) 4 y and 6 mo; median 7 y 2 mo; interquartile range (IQR) 3 y 9 mo–11 y 4 mo); 38.7%, (*n* = 1331/3441) female.

### 3.1. Rehabilitation Status

Nearly half (48.3%, *n* = 1653/3422) of the registered children with CP from all four countries had never received any type of rehabilitation services. However, the proportion of children who never received rehabilitation did vary significantly across countries, with 45.8% (*n* = 82/182) in Nepal up to 66.2% (*n* = 86/130) in Indonesia in the population-based settings (*p* = 0.001) and 26.7% (*n* = 77/277) in Ghana. Of those who received rehabilitation services, the majority received physical therapy (ranging between 69.8–90.0% in population-based settings and 98.0% in institution-based settings, i.e., Ghana). Access to assistive devices was low (6.3–16.3%) in Bangladesh, Indonesia, and Nepal, whereas, in Ghana, more than a third of children had received an assistive device. The mean (SD) age of commencing rehabilitation services substantially differed between the countries in population-based settings, ranging from 2.9 (3.0) y in Indonesia and 3.9 (3.1) y in Nepal. Most of the children received services between age 0 y and 4 y in both population-based (i.e., Bangladesh, Indonesia, and Nepal) and institution-based settings. Hospital/health centre were the primary rehabilitation services provider in Nepal (77.2%, *n* = 71/92), Indonesia (43.9%, *n* = 18/41), and Ghana (97.9%, *n* = 191/195), while non-government organisation was the commonest service provider in Bangladesh (45.1%, *n* = 626/1387). Lack of awareness regarding service needs and availability was the most commonly reported reason for not receiving rehabilitation in all four countries (84.2% (*n* = 1141/1355) in Bangladesh, 71.4% (*n* = 45/63) in Nepal, 70.9% (*n* = v61/86) in Indonesia, and 68.9% (*n* = 51/74) in Ghana) (Table 1).

### 3.2. Factors Influencing Rehabilitation Services Utilisation among Children with CP

#### 3.2.1. Age and Sex of Children with CP

The proportion of children who received rehabilitation services gradually declined as age increased in Bangladesh, Indonesia, and Nepal. However, the findings were contradictory in Ghana (Table 2).

Rehabilitation service utilisation was proportionally higher among male children compared to their female peers in all four countries. However, this difference was only significant in Bangladesh (*p* = 0.013) (Table 2).

#### 3.2.2. Education of Parents

Higher level of maternal education (secondary education and above) was significantly associated with accessing rehabilitation services in all countries (*p* < 0.05) except Ghana. Similar findings were also found for the father’s education (Table 2).

#### 3.2.3. Monthly Family Income

Children with CP from families with a monthly income of USD above 100 had the highest rehabilitation service utilisation while this proportion was lowest among families with a monthly income of USD 0–50 or 51–100 in all four countries (Table 2).

#### 3.2.4. Predominant Motor Type and Topography of CP

Across the predominant motor types of CP, rehabilitation services utilisation was highest among children with dyskinesia in Bangladesh and Nepal. In contrast, children with a spastic motor type had superior rehabilitation service utilisation in Indonesia and Ghana. In terms of spastic topography, the proportion of children receiving rehabilitation was highest among children with spastic tri/quadriplegia in all four countries (Table 3).

#### 3.2.5. GMFCS and MACS Level

A substantially higher number of children with GMFCS level III–V received rehabilitation services compared to those with GMFCS level I–II in all four countries. Children with MACS level III–V had a significantly higher proportion of rehabilitation service utilisation compared to those with MACS level I–II in Bangladesh and Nepal (Table 3).

#### 3.2.6. Associated Impairments

The utilisation of rehabilitation services was significantly higher among children with epilepsy (*p* = 0.019) and speech impairment (*p* = 0.032) in Ghana and children with hearing impairment (*p* = 0.016) in Indonesia. Comparatively, this rate was significantly lower for intellectual impairment (*p* = 0.020), visual impairment (*p* = 0.004), and hearing impairment (*p* < 0.001) in Bangladesh and for intellectual impairment (*p* = 0.006) in Nepal. The cohort of children with 3–5 associated impairments had significantly lower rehabilitation service utilisation in Bangladesh (*p* < 0.001) (Table 3).

#### 3.2.7. Age of CP Diagnosis

The mean (SD) age of CP diagnosis was significantly lower among children who received rehabilitation compared to those who never received rehabilitation in Bangladesh (3 (3) y vs. 5 (4) y, *p* < 0.001, respectively) and Indonesia (3 (3) y vs. 8 (5) y, *p* < 0.001, respectively) (Table 3).

#### 3.2.8. Motor Severity and Rehabilitation Service Utilisation

To assess the relationship between motor severity and rehabilitation service utilisation among children registered in the GLM-CPR, clinical characteristics of children with CP were assessed according to the GMFCS level I–II and GMFCS level III–V distinctly. Overall, children with GMFCS level I–II having dyskinetic or ataxic type of CP, tri/quadriplegia topography, MACS level III–V, 1–2 associated impairments, and early diagnosis had higher rehabilitation service utilisation. On the other hand, children with GMFCS level III–V having a dyskinetic type of CP, tri/quadriplegia topography, MACS level III–V, no associated impairments, and early diagnosis had greater rehabilitation service utilisation (Table 4).

### 3.3. Predictors of Not Receiving Rehabilitation Services among Children with CP

The adjusted analysis shows that female children, illiterate and primary level completed mothers, illiterate fathers, monthly family income of USD 51–100, GMFCS level III–V, and hearing impairment significantly predict the rehabilitation service utilisation of children in Bangladesh. In comparison, intellectual impairment was significantly associated with rehabilitation service utilisation in Nepal. Amongst the Indonesian cohort, primary educated mothers and hearing impairment were found to significantly influencing rehabilitation service utilisation. In Ghana, 5–9 y age group and 10–14 y age group were significantly associated with rehabilitation service utilisation (Table 5).

## 4. Discussion

This is one of the first studies reporting rehabilitation status and cross-cultural comparison of predictors of rehabilitation service utilisation among children with CP in four LMICs, i.e., Bangladesh, Nepal, Indonesia, and Ghana. Our study revealed that nearly half of the children with CP registered into the GLM-CPR had never received rehabilitation services in their lifetime, and the age of commencing rehabilitation was substantially delayed in all four countries. The study findings also suggest that the predictors of rehabilitation service utilisation vary between the studied countries, which could be partially attributed to the different cohort sizes of the CP registers included in GLM-CPR.

The proportion of children receiving rehabilitation declined with increasing age in all studied countries except Ghana, which is most likely because of the institution-based recruitment (i.e., selection bias). Young age has been reported as a positive predictor of rehabilitation service utilisation in earlier studies [14,22]. However, there is an acute shortage of rehabilitation workforce and facilities in LMICs, which acts as a key access barrier for children with CP [23]. For instance, only seven rehabilitation facilities are available for 1 million people and only 6% of them are established in rural settings in Bangladesh [24]. A recent study argued that the establishment of surveillance programs and rehabilitation facilities enables the early initiation of rehabilitation services [25]. Hence, an emphasis on training, recruitment, and distribution of rehabilitation professionals and allocation of resources for the establishment of active surveillance programs and rehabilitation facilities is crucial for LMICs in order to improve access to rehabilitation.

In terms of gender, female children with CP had a lower likelihood of receiving rehabilitation services when compared to male children in Bangladesh. Nuri et al. [26] described a similar finding in terms of access to services among children with CP in Bangladesh, whereas Sinha and Sharma [10] reported no relationship between sex and rehabilitation service utilisation in India. Women with disabilities may face a double burden, because of their gender roles and disabilities, in LMICs [27]. McConachie et al. [28] described that having a male child can influence parents to seek rehabilitation services, particularly in rural settings. This might be related to the notion that male children need to be able to support a family in the future. Health care professionals, therefore, should be aware of this sex-related barrier of rehabilitation and counsel and encourage parents to access rehabilitation services for their children without considering their gender identity. This information can also inform female empowerment campaigns of government and non-government organisations to improve access of female children with disabilities to rehabilitation services.

We observed that children of illiterate and primary educated parents, mainly mothers, in Bangladesh and Indonesia had a significantly lower likelihood of receiving rehabilitation. These findings are in line with earlier studies reporting a significant relationship between parental education and the exposure of physiotherapy service in the USA [15] and India [10]. Illiterate and poorly educated families are likely to have low socio-economic status, limited time because of their daily jobs, and live further away from cities where more services exist. Children from impoverished families were less likely to receive rehabilitation services in Bangladesh. Poverty as a key barrier to the rehabilitation of children with CP has been recognised in other LMICs and in HICs previously [9,10,15,29]. McConachie et al. [28] reported that a majority of children with CP cannot access rehabilitation services because of the costs associated with rehabilitation services in less-resourced settings. Khandaker et al. [3] ascertained that more than 75% of children with CP are from ultra-poor families in Bangladesh. A sustainable early intervention and rehabilitation service delivery model for children with disabilities, incorporating free services for poor families and nominal fees for families who are able to pay, is essential, particularly for rural areas. In this service delivery model, the cost of the services might be defined based on the financial capacity of clients while ensuring that no one forgoes rehabilitation because of financial constraints. Government, non-government organisations, and private entrepreneurs should come forward to establish a sustainable model of rehabilitation services in rural areas to help underprivileged families to access rehabilitation services [30].

Amongst the clinical predictors, children with GMFCS level III–V and MACS level III-V had a significantly higher likelihood of receiving rehabilitation services in Bangladesh and Nepal, respectively. These findings are in agreement with studies conducted in Canada [12], the USA [16], the Netherlands [31], and Australia [14]. It is most likely that parents of children with GMFCS level III–V or MACS level III–V were more concerned about rehabilitation as their children developed visible severe functional impairment and required greater assistance in activities of daily living. Recent evidence suggests that early initiation of rehabilitation focusing on goal-directed activity-focused training can help to prevent future functional impairments, and this includes children in GMFCS I and II [32].

The chance of receiving rehabilitation was significantly lower among children with hearing and intellectual impairments in Bangladesh and Nepal, respectively. In Indonesia, however, children who had hearing impairment were more likely to receive rehabilitation services. Children with 1–2 impairments had a higher chance of receiving rehabilitation services compared to children with no associated impairments in Nepal. Like ours, a contradictory finding on the association between associated impairments and rehabilitation service use was reported in earlier studies. Liljenquist et al. [15] reported that rehabilitation service utilisation is higher among children with associated impairments in the USA. In contrast, Majnemer et al. [13] observed a substantially higher utilisation of rehabilitation among children with intellectual impairments in Canada. No definite conclusion can be drawn from these contrasting findings. Further study with open-ended questions is required to explore in-depth information on the relationship between associated impairments and rehabilitation service utilisation. In the interim, health professionals should be trained on the management of associated impairments of CP and to support families to access rehabilitation services regardless of the absence or presence of associated impairments.

Our study findings identified that most of the participating children in LMICs are diagnosed at an age when they are past the period for maximum neuroplasticity. On a positive note, the study also indicates that children who were diagnosed at an early age had a significantly higher probability of receiving rehabilitation services. These findings, once again, underscore the necessity of establishing early diagnostic and intervention facilities to improve access to rehabilitation services for children with CP in LMICs.

Inadequate access to rehabilitation services is a major concern in low-resource settings [25,33]. In resource-limited settings, like our study sites, there is already a lack of institutional rehabilitation services to cater for the unmet service need. On top of that, in our study, we observed that most of the primary caregivers were unaware of the service needs for their children and its availability. In such settings, community-based rehabilitation services along with advocacy or educational programs could raise awareness of the primary caregivers and thus improve service accessibility and utilisation for their children with CP. In a recent study of ours, we have observed that operating community-based rehabilitation centres as part of an active population-based surveillance system enhanced the caregiver awareness substantially, enabling early diagnosis and early rehabilitation service use among children with CP in Bangladesh [25].

Despite our extensive effort, there are some limitations in this study. (i) The number of children with CP included in this study from four countries varied largely due to different timepoint of the establishment of registers (e.g., BCPR in 2015, community-based survey in Indonesia in 2017, NCPR in 2018, and GCPR in 2018). However, over time, these CP registers and their cohort size would mature. (ii) Additionally, the type of surveillance mechanisms (i.e., population-based and institution-based) might have impacted the findings of the study. For instance, institution/hospital-based surveillance systems are likely to overestimate the service access, while community-based surveillance in a rural setting may slightly underestimate service access. Despite these challenges, these CP registers are an important and essential source of information to better understand CP and service use in these developing countries—by bringing these data together, this innovative program provides us with a snapshot of CP in LMICs that would not otherwise be possible. (iii) The findings on whether children received rehabilitation reported in this study might be an overestimation of the true rehabilitation status, as data presented here reflects the proportion of children with CP who have ever received any rehabilitation service with a minimum of one session to be considered as a recipient of rehabilitation services. (iv) The KIM method has a 77.6% case ascertainment rate which indicates that there might be incomplete case ascertainment in this study, particularly for the milder form of CP [21]. However, the KIM has been found to be a highly cost-effective method to recruit children with disabilities from the community in low-resource settings like LMICs. (v) Responses of primary caregivers were considered for assessment of the rehabilitation status and presence of associated impairments. Therefore, the risk of recall bias could not be excluded.

## 5. Conclusions

Nearly half the children with CP do not have access to rehabilitation services in Bangladesh, Nepal, and Indonesia. Although the rate of a severe form of motor severity (i.e., GMFCS level III–V) was higher among the participating children, the utilisation of assistive devices is extremely poor in Bangladesh, Nepal, and Indonesia. Additionally, the age of rehabilitation service commencement is noticeably delayed, limiting the opportunity for neuroplasticity as well as functional recovery. To enable early intervention uptake and prevent functional limitations, the establishment of early diagnostic facilities is essential. This study revealed some socio-demographic (i.e., age and sex of children, parental education, and monthly family income) and clinical factors (i.e., GMFCS level, MACS level, and associated impairments) that were significantly associated with rehabilitation service utilisation. Our study highlights that strong advocacy programs and policy-level work are essential to raise awareness for early childhood intervention and establish parents-led community-based rehabilitation facilities in LMICs. Policymakers and service providers could use these findings to increase access to rehabilitation, and improve equity in rehabilitation service utilisation for better functional outcomes of children with CP in these LMIC settings.

## Figures and Tables

**Table 1:** Rehabilitation status of children with CP in Bangladesh, Nepal, Indonesia, and Ghana.

Characteristics	Population-Based			*p* Value	Institution-Based
	Bangladesh *n* (%)	Nepal *n* (%)	Indonesia *n* (%)		Ghana *n* (%)
	*n* = 2852	*n* = 182	*n* = 130		*n* = 277
Ever received rehabilitation service	*n* = 2836 ^1^	*n* = 179 ^1^	*n* = 130		*n* = 277
No	1411 (49.8)	82 (45.8)	86 (66.2)	0.001 ^3^	74 (26.7)
Yes	1425 (50.2)	97 (54.2)	44 (33.8)		203 (73.3)
Type of rehabilitation service received	*n* = 1404 ^1,2^	*n* = 96 ^1,2^	*n* = 43 ^1,2^		*n* = 203 ^2^
Physical therapy	1264 (90.0)	67 (69.8)	37 (86.0)	<0.001 ^3^	200 (98.0)
Assistive device	124 (8.8)	6 (6.3)	7 (16.3)		102 (50.0)
Advice	156 (11.1)	22 (22.9)	6 (14.0)		2 (1.0)
Surgery	0 (0.0)	10 (10.4)	1 (2.3)		0 (0.0)
Primary location of rehabilitation service received	*n* = 1387 ^1^	*n* = 92 ^1^	*n* = 41 ^1^		*n* = 195 ^1^
NGO centre	626 (45.1)	9 (9.8)	1 (17.0)	<0.001 ^3^	2 (1.0)
Hospital/health care centre	443 (31.9)	71 (77.2)	18 (43.9)		191 (97.9)
Home based	153 (11.0)	6 (6.5)	14 (34.1)		0 (0.0)
Private clinic	141 (10.2)	6 (6.5)	2 (5.0)		2 (1.0)
Special school	24 (1.7)	0 (0.0)	0 (0.0)		0 (0.0)
Age of first commencement of rehabilitation service (in years)	*n* = 1365 ^1^	*n* = 87 ^1^	*n* = 43 ^1^		*n* = 202 ^1^
Mean (SD)	3.8 (3.1)	3.9 (3.1)	2.9 (2.6)	0.184 ^4^	3.0 (1.8)
Median (IQR)	3.0 (1.5–5.0)	3.0 (1.5–5.1)	1.0 (2.0–4.0)	0.144 ^5^	1.5 (2.5–4.0)
0–4	950 (69.6)	55 (63.2)	36 (83.7)	0.154 ^3^	169 (83.7)
5–9	324 (23.7)	25 (28.7)	4 (9.3)		32 (15.8)
10 and above	91 (6.7)	7 (8.0)	3 (7.0)		1 (0.5)
Reason for not receiving rehabilitation services	*n* = 1355 ^1^	*n* = 63 ^1^	*n* = 86		*n* = 74
Lack of awareness	1141 (84.2)	45 (71.4)	61 (70.9)	<0.001 ^3^	51 (68.9)
Financial constraint	185 (13.7)	12 (19.0)	13 (15.1)		20 (27.0)
Transport problem	21 (1.5)	1 (1.6)	9 (10.5)		1 (1.4)
Others ^6^	8 (0.6)	5 (7.9)	3 (3.5)		2 (2.7)

^1^ Missing value exists; ^2^ Not mutually exclusive; ^3^ Chi-squared test; ^4^ ANOVA test; ^5^ Kruskal–Wallis H test; ^6^ Others include personal problems, lost hope, lack of service, doctors did not offer advice, lack of time, parents refused.

**Table 2:** Distribution of socio-demographic factors according to rehabilitation status of children with CP in Bangladesh, Nepal, Indonesia, and Ghana.

Socio-Demographic Characteristics	Ever Received Any Rehabilitation Services											
	Population-Based									Institution-Based		
	Bangladesh			Nepal			Indonesia			Ghana		
	*n* = 2852			*n* = 182			*n* = 130			*n* = 277		
	No *n* (Row%/Column%)	Yes *n* (Row%/Column%)	*p* Value	No *n* (Row%/Column%)	Yes *n* (Row%/Column%)	*p* Value	No *n* (Row%/Column%)	Yes *n* (Row%/Column%)	*p* Value	No *n* (Row%/Column%)	Yes *n* (Row%/Column%)	*p* Value
Age (in years)	*n* = 2799 ^1^			*n* = 177 ^1^			*n* = 128 ^1^			*n* = 261 ^1^		
0–4	432 (46/31)	507 (54/36)	0.001 ^2^	9 (39/11)	14 (61/15)	0.851 ^2^	22 (63/26)	13 (37/30)	0.798 ^2^	40 (45/64)	49 (55/25)	<0.001 ^2^
5–9	466 (48/34)	502 (52/36)		28 (44/35)	36 (56/38)		28 (67/33)	14 (33/33)		14 (14/23)	88 (86/44)	
10–14	331 (54/24)	288 (46/20)		25 (47/32)	28 (53/29)		22 (65/26)	12 (35/28)		8 (16/13)	43 (84/22)	
15–18	150 (58/11)	107 (42/8)		17 (50/22)	17 (50/18)		13 (77/15)	4 (23/9)		0 (0/0)	19 (100/9)	
Sex	*n* = 2852			*n* = 181 ^1^			*n* = 130			*n* = 277 ^1^		
Male	837 (48/59)	910 (52/64)	0.013 ^2^	47 (42/57)	64 (58/67)	0.199 ^2^	43 (59/50)	30 (41/68)	0.048 ^2^	42 (25/57)	126 (75/62)	0.423 ^2^
Female	574 (53/41)	515 (47/36)		35 (52/43)	32 (48/33)		43 (75/50)	14 (25/32)		32 (29/43)	77 (71/38)	
Education of mother	*n* = 2845 ^1^			*n* = 170 ^1^			*n* = 130			*n* = 277		
No education	534 (63/38)	316 (37/22)	<0.001 ^2^	41 (62/52)	25 (38/28)	0.007 ^2^	10 (63/12)	6 (37/14)	0.013 ^2^	32 (28/43)	81 (72/40)	0.774 ^2^
Primary	592 (53/42)	533 (47/38)		20 (36/25)	36 (64/40)		50 (78/58)	14 (22/32)		25 (27/34)	67 (73/33)	
Secondary and above	282 (33/20)	572 (67/40)		18 (39/23)	28 (61/32)		26 (52/30)	24 (48/54)		17 (24/23)	55 (76/27)	
Education of father	*n* = 2827 ^1^			*n* = 167 ^1^			*n* = 124 ^1^			*n* = 277		
No education	673 (61/48)	430 (39/30)	<0.001 ^2^	24 (67/31)	12 (33/14)	0.030 ^2^	15 (75/18)	5 (25/12)	0.405 ^2^	22 (32/30)	46 (68/22)	0.320 ^2^
Primary	428 (49/31)	448 (51/32)		30 (43/38)	40 (57/46)		41 (68/50)	19 (32/45)		30 (27/40)	79 (73/40)	
Secondary and above	301 (36/21)	531 (64/38)		24 (41/31)	35 (59/40)		26 (59/32)	18 (41/43)		22 (22/30)	78 (78/38)	
Monthly family income (in USD) ^3^	*n* = 2828 ^1^			*n* = 182			*n* = 130			*n* = 277		
0–50	97 (50/7)	99 (50/7)	<0.001 ^2^	18 (58/22)	13 (42/13)	0.278 ^2^	74 (71/86)	30 (29/68)	0.012 ^2^	41 (31/55)	90 (69/44)	0.261 ^2^
51–100	871 (56/62)	680 (44/48)		28 (46/34)	33 (54/34)		9 (60/11)	6 (40/14)		17 (22/23)	60 (78/30)	
Above 100	432 (41/31)	633 (59/45)		36 (41/44)	51 (59/53)		3 (27/3)	8 (73/18)		16 (23/22)	53 (77/26)	
Median (IQR) monthly family income (in USD)	83 (71–119)	95 (71–135)	<0.001 ^4^	90 (61–167)	108 (72–180)	0.087 ^4^	23 (14–35)	30 (14–70)	0.432 ^4^	37 (19–93)	56 (28–111)	0.056 ^4^

^1^ Missing value exists; ^2^ Chi-squared test; ^3^ 1 USD ≈ 84.43 Bangladeshi taka/111 Nepalese rupee/14296 Indonesian rupee/5.4 Cedi; ^4^ Mann–Whitney U test.

**Table 3:** Distribution of clinical factors according to rehabilitation status of children with CP in Bangladesh, Nepal, Indonesia, and Ghana.

Clinical Characteristic	Ever Received Any Rehabilitation Services											
	Population-Based									Institution-Based		
	Bangladesh			Nepal			Indonesia			Ghana		
	*n* = 2852			*n* = 182			*n* = 130			*n* = 277		
	No *n* (Row%/Column%)	Yes *n* (Row%/Column%)	*p* Value	No *n* (Row%/Column%)	Yes *n* (Row%/Column%)	*p* Value	No *n* (Row%/Column%)	Yes *n* (Row%/Column%)	*p* Value	No *n* (Row%/Column%)	Yes *n* (Row%/Column%)	*p* Value
Type of CP	*n* = 2852			*n* = 182			*n* = 130			*n* = 277		
Spastic	1129 (49/80)	1155 (51/81)	0.401 ^2^	65 (47/79)	74 (53/76)	0.964 ^3^	65 (62/76)	40 (38/91)	0.151 ^3^	51 (24/69)	166 (76/82)	0.040 ^2^
Dyskinetic	84 (48/6)	92 (52/6)		2 (33/2)	4 (67/4)		10 (77/12)	3 (23/7)		4 (57/5)	3 (43/1)	
Ataxic	51 (58/4)	37 (42/3)		7 (44/9)	9 (56/9)		1 (100/1)	0 (0/0)		8 (29/11)	20 (71/10)	
Hypotonic	147 (51/10)	141 (49/10)		8 (44/10)	10 (56/10)		10 (91/11)	1 (9/2)		11 (44/15)	14 (56/7)	
Spastic topography	*n* = 2293			*n* = 141			*n* = 105			*n* = 217		
Mono/hemiplegia	350 (55/31)	287 (45/25)	<0.001 ^2^	29 (54/45)	25 (46/34)	0.204 ^2^	10 (71/15)	4 (29/10)	0.724 ^2^	13 (38/26)	21 (62/13)	0.063 ^2^
Diplegia	219 (53/19)	191 (47/16)		8 (57/12)	6 (43/8)		13 (62/20)	8 (38/20)		12 (25/23)	36 (75/22)	
Tri/quadriplegia	560 (45/50)	677 (55/59)		28 (39/43)	43 (61/58)		42 (60/65)	28 (40/70)		26 (19/51)	109 (81/65)	
GMFCS levels	*n* = 2836 ^1^			*n* = 182			*n* = 130			*n* = 277		
I–II	420 (57/30)	321 (43/23)	<0.001 ^2^	42 (53/51)	38 (47/39)	0.106 ^2^	17 (81/20)	4 (19/9)	0.118 ^2^	20 (33/27)	41 (67/20)	0.225 ^2^
III–V	988 (48/70)	1092 (52/77)		40 (40/49)	59 (60/61)		69 (63/80)	40 (37/91)		54 (25/73)	162 (75/80)	
MACS level ^4^	*n* = 2220 ^1^			*n* = 159 ^1^			*n* = 111			*n* = 0 ^5^		
I–II	346 (50/35)	346 (50/29)	0.003 ^2^	46 (54/64)	40 (46/48)	0.042 ^2^	29 (71/40)	12 (29/32)	0.399 ^2^	n/a	n/a	n/a
III–V	654 (43/65)	861 (57/71)		26 (37/36)	44 (63/52)		44 (63/60)	26 (37/68)		n/a	n/a	
**Type of Associated Impairment**												
Epilepsy	*n* = 2835 ^1^			*n* = 182			*n* = 123 ^1^			*n* = 277		
Yes	428 (48/31)	464 (52/33)	0.197 ^2^	24 (48/29)	26 (52/27)	0.714 ^2^	8 (53/10)	7 (47/17)	0.242 ^2^	4 (11/5)	33 (89/16)	0.019 ^2^
No	975 (51/69)	952 (49/67)		58 (45/71)	71 (55/73)		74 (69/90)	34 (31/83)		70 (29/95)	170 (71/84)	
Intellectual	*n* = 1944 ^1^			*n* = 132 ^1^			*n* = 63 ^1^			*n* = 96 ^1^		
Yes	570 (53/58)	497 (47/53)	0.020 ^2^	45 (54/78)	39 (46/54)	0.006 ^2^	24 (63/56)	14 (37/70)	0.284 ^2^	13 (19/62)	55 (81/73)	0.308 ^2^
No	417 (48/42)	450 (52/47)		13 (28/22)	33 (72/46)		19 (76/44)	6 (24/30)		8 (29/38)	20 (71/27)	
Visual	*n* = 2813 ^1^			*n* = 179 ^1^			*n* = 130			*n* = 277		
Yes	257 (56/18)	201 (44/14)	0.004 ^2^	9 (50/11)	9 (50/9)	0.683 ^2^	10 (56/12)	8 (44/18)	0.306 ^2^	5 (24/7)	16 (76/8)	0.754 ^2^
No	1141 (49/82)	1198 (51/86)		71 (45/89)	87 (55/91)		76 (68/88)	36 (32/82)		69 (27/93)	187 (73/92)	
Hearing	*n* = 2835 ^1^			*n* = 179 ^1^			*n* = 130			*n* = 276 ^1^		
Yes	364 (63/26)	212 (37/15)	<0.001 ^2^	17 (47/21)	19 (53/20)	0.871 ^2^	12 (46/14)	14 (54/32)	0.016 ^2^	18 (23/25)	60 (77/30)	0.425 ^2^
No	1042 (47/74)	1201 (53/85)		64 (46/79)	76 (54/80)		74 (71/86)	30 (29/68)		55 (28/75)	143 (72/70)	
Speech	*n* = 2834 ^1^			*n* = 181 ^1^			*n* = 129 ^1^			*n* = 273 ^1^		
Yes	1054 (50/75)	1066 (50/76)	0.744 ^2^	65 (46/80)	78 (54/80)	0.978 ^2^	62 (62/73)	38 (38/86)	0.083 ^2^	58 (24/82)	184 (76/91)	0.032 ^2^
No	352 (50/25)	346 (50/24)		16 (46/20)	19 (54/20)		23 (79/27)	6 (21/14)		13 (42/18)	18 (58/9)	
Number of Associated impairments	*n* = 1889 ^1^			*n* = 130 ^1^			*n* = 60 ^1^			*n* = 95 ^1^		
None	212 (52/22)	198 (48/22)	<0.001 ^2^	9 (47/15)	10 (53/14)	0.077 ^2^	13 (81/32)	3 (19/16)	0.416 ^2^	2 (18/10)	9 (82/12)	0.268 ^2^
1–2	406 (47/42)	460 (53/50)		23 (36/40)	41 (64/59)		20 (63/49)	12 (37/63)		10 (30/50)	23 (70/31)	
3–5	349 (58/36)	254 (42/28)		26 (58/45)	19 (42/27)		8 (67/19)	4 (33/21)		8 (16/40)	43 (84/57)	
Age of CP diagnosis	*n* = 2787 ^1^			*n* = 169 ^1^			*n* = 128 ^1^			*n* = 277		
Mean (SD)	5 (4)	3 (3)	<0.001 ^6^	5 (4)	4 (5)	0.700 ^6^	8 (5)	3 (3)	<0.001 ^6^	3 (2)	3 (2)	0.847 ^6^

^1^ Missing value exists; ^2^ Chi-squared test; ^3^ Fisher’s Exact test; ^4^ MACS was assessed among children aged at or over four years; ^5^ MACS data for Ghana were not available; ^6^ Independent sample *t* test; n/a = data not available.

**Table 4:** Distribution of clinical factors of children with CP according to their GMFCS level and rehabilitation status in Bangladesh, Nepal, Indonesia, and Ghana.

Clinical Characteristics	GMFCS Level I–II								GMFCS Level III–V							
	Bangladesh		Nepal		Indonesia		Ghana		Bangladesh		Nepal		Indonesia		Ghana	
	Ever Received Rehabilitation		Ever Received Rehabilitation		Ever Received Rehabilitation		Ever Received Rehabilitation		Ever Received Rehabilitation		Ever Received Rehabilitation		Ever Received Rehabilitation		Ever Received Rehabilitation	
	No *n* (%) ^3^	Yes *n* (%) ^3^	No *n* (%) ^3^	Yes *n* (%) ^3^	No *n* (%) ^3^	Yes *n* (%) ^3^	No *n* (%) ^3^	Yes *n* (%) ^3^	No *n* (%) ^3^	Yes *n* (%) ^3^	No *n* (%) ^3^	Yes *n* (%) ^3^	No *n* (%) ^3^	Yes *n* (%) ^3^	No *n* (%) ^3^	Yes *n* (%) ^3^
Predominant Motor Type ^1^																
Spastic	336 (56.3)	261 (43.7)	32 (53.3)	28 (46.7)	12 (75.0)	4 (25.0)	10 (32.3)	21 (67.7)	791 (47.2)	886 (52.8)	33 (41.8)	46 (58.2)	53 (59.6)	36 (40.4)	41 (22.0)	145 (78.0)
Dyskinetic	23 (52.3)	21 (47.7)	2 (50.0)	2 (50.0)	2 (100.0)	0 (0.0)	1 (100.0)	0 (0.0)	61 (46.6)	70 (53.4)	0 (0.0)	2 (100.0)	8 (72.7)	3 (27.3)	3 (50.0)	3 (50.0)
Ataxia	27 (60.0)	18 (40.0)	7 (58.3)	5 (41.7)	1 (100.0)	0 (0.0)	8 (28.6)	20 (71.4)	24 (55.8)	19 (44.2)	0 (0.0)	4 (100.0)	0 (0.0)	0 (0.0)	0 (0.0)	0 (0.0)
Hypotonia	34 (61.8)	21 (38.2)	1 (25.0)	3 (75.0)	2 (100.0)	0 (0.0)	1 (100.0)	0 (0.0)	112 (48.9)	117 (51.1)	7 (50.0)	7 (50.0)	8 (88.9)	1 (11.1)	10 (41.7)	14 (58.3) ^6^
Topography ^1^																
Mono/hemiplegia	235 (57.2)	176 (42.8)	23 (53.5)	20 (46.5)	7 (77.8)	2 (22.2)	8 (30.8)	18 (69.2)	113 (51.6)	106 (48.4)	6 (54.5)	5 (45.5)	3 (60.0)	2 (40.0)	5 (62.5)	3 (37.5)
Diplegia	69 (59.5)	47 (40.5)	4 (66.7)	2 (33.3)	3 (100.0)	0 (0.0)	1 (50.0)	1 (50.0)	150 (51.4)	142 (48.6)	4 (50.0)	4 (50.0)	10 (55.6)	8 (44.4)	11 (23.9)	35 (76.1)
Tri/quadriplegia	32 (45.7)	38 (54.3)	5 (45.5)	6 (54.5)	2 (50.0)	2 (50.0)	1 (33.3)	2 (66.7)	528 (45.3)	638 (54.7)	23 (38.3)	37 (61.7)	40 (60.6)	26 (39.4)	25 (18.9)	107 (81.1) ^6^
MACS Level ^1,4^																
I–II	246 (55.5)	197 (44.5)	36 (54.5)	30 (45.5)	12 (80.0)	3 (20.0)	n/a	n/a	100 (40.7)	146 (59.3)	10 (50.0)	10 (50.0)	17 (65.4)	9 (34.6)	n/a	n/a
III–V	87 (49.2)	90 (50.8)	2 (28.6)	5 (71.4)	1 (50.0)	1 (50.0)	n/a	n/a	566 (42.4)	769 (57.6)	24 (38.1)	39 (61.9)	43 (63.2)	25 (36.8)	n/a	n/a
Number of Associated Impairments **^1^**																
None	110 (60.8)	71 (39.2)	8 (66.7)	4 (33.3)	1 (100.0)	0 (0.0)	1 (33.3)	2 (66.7)	102 (45.1)	124 (54.9)	1 (14.3)	6 (85.7)	12 (80.0)	3 (20.0)	1 (12.5)	7 (87.5)
1–2 impairments	168 (54.2)	142 (45.8)	13 (39.4)	20 (60.6)	7 (87.5)	1 (12.5)	7 (38.9)	11 (61.1)	237 (42.9)	315 (57.1)	10 (32.3)	21 (67.7)	13 (54.2)	1 (45.8)	3 (20.0)	12 (80.0)
3–5 impairments	45 (54.2)	38 (45.8)	8 (57.1)	6 (42.9)	1 (100.0)	0 (0.0)	2 (25.0)	6 (75.0)	304 (58.5)	216 (41.5) ^5^	18 (58.1)	13 (41.9) ^6^	7 (63.6)	4 (36.4)	6 (14.0)	37 (86.0)
Mean (SD) age of CP diagnosis ^2^	5.7 (4.6)	3.6 (3.8) ^5^	6.1 (4.9)	5.5 (4.7)	9.7 (5.0)	3.3 (1.3) ^6^	4.0 (3.0)	3.1 (2.0)	4.5 (4.3)	2.8 (3.1) ^5^	3.2 (3.1)	3.6 (4.2)	7.6 (4.5)	3.2 (2.9) ^5^	2.0 (1.7)	2.5 (1.7)

^1^ Chi-squared test; ^2^ Independent sample *t* test; ^3^ row percentages presented; ^4^ MACS data for Ghana were not available; ^5^ *p* < 0.001; ^6^ *p* < 0.05; n/a = data not available.

**Table 5:** Socio-demographic and clinical predictors of not receiving rehabilitation services among children with CP in Bangladesh, Nepal, Indonesia, and Ghana.

Characteristics	Not Receiving Rehabilitation Services							
	Population-Based						Institution-Based	
	Bangladesh		Nepal		Indonesia		Ghana	
	*n* = 2852		*n* = 182		*n* = 130		*n* = 277	
	Unadjusted OR (CI)	Adjusted OR (CI) ^5^	Unadjusted OR (CI)	Adjusted OR (CI) ^5^	Unadjusted OR (CI)	Adjusted OR (CI) ^5^	Unadjusted OR (CI)	Adjusted OR (CI) ^5^
Age (in years)								
0–4	Ref							
5–9	1.1 (0.9–1.3)	0.9 (0.6–1.3)	1.2 (0.5–3.2)		1.2 (0.5–3.0)		0.2 (0.1–0.4) ^1^	0.2 (0.1–0.5) ^1^
10–14	1.3 (1.1–1.7) ^2^	1.1 (0.7–1.6)	1.4 (0.5–3.8)		1.1 (0.4–2.9)		0.2 (0.2–1.2) ^2^	0.3 (0.1–0.6) ^2^
15–18	1.6 (1.2–2.2) ^1^	1.4 (0.9–2.3)	1.6 (0.5–4.6)		1.9 (0.5–7.1)		n/a ^6^	n/a ^6^
Sex								
Male	Ref							
Female	1.2 (1.0–1.4) ^2^	1.3 (1.0–1.7) ^2^	1.5 (0.8–2.7)		2.1 (1.0–4.6)		1.2 (0.7–2.1)	
Education of mother								
No education	3.4 (2.8–4.2) ^1^	2.1 (1.4–3.1) ^1^	2.6 (1.2–5.5) ^2^	2.6 (0.6–12.2)	1.5 (0.5–4.9)	1.3 (0.4–4.4)	1.3 (0.6–2.5)	
Primary	2.3 (1.9–2.7) ^1^	1.5 (1.1–2.1) ^2^	0.9 (0.4–1.9)	0.9 (0.2–3.5)	3.3 (1.5–7.4) ^2^	2.7 (1.1–6.5) ^2^	1.2 (0.6–2.5)	
Secondary and above	Ref							
Education of father								
No education	2.8 (2.3–3.3) ^1^	1.9 (1.3–2.7) ^2^	2.9 (1.2–6.9) ^2^	1.3 (0.3–6.6)	2.1 (0.6–6.7)		1.7 (0.8–3.4)	
Primary	1.7 (1.4–2.0) ^1^	1.2 (0.9–1.7)	1.1 (0.5–2.2)	0.6 (0.2–2.1)	1.5 (0.7–3.4)		1.3 (0.7–2.5)	
Secondary and above	Ref							
Monthly family income (in USD)								
0–50	1.4 (1.1–1.9) ^2^	1.5 (0.9–2.4)	2.0 (0.9–4.5)		6.6 (1.6–26.5) ^2^	3.9 (0.9–17.5)	1.5 (0.8–2.9)	
51–100	1.9 (1.6–2.2) ^1^	1.9 (1.4–2.4) ^1^	1.2 (0.6–2.3)		4.0 (0.7–21.5)	2.7 (0.5–16.1)	0.9 (0.4–2.0)	
Above 100	Ref							
Type of CP								
Spastic	Ref							
Dyskinetic	0.9 (0.7–1.3)		0.6 (0.1–3.2)		2.1 (0.5–7.9)		4.3 (0.9–20.0)	
Ataxic	1.4 (0.9–2.2)		0.9 (0.3–2.5)		n/a ^6^		1.3 (0.5–3.1)	
Hypotonic	1.1 (0.8–1.4)		0.9 (0.3–2.4)		6.2 (0.8–49.9)		2.6 (1.1–6.0) ^2^	
Topography								
Mono/hemiplegia	Ref							
Diplegia	0..9 (0.7–1.2)	1.3 (0.9–1.9)	1.1 (0.4–3.8)		0.7 (0.2–2.8)		0.5 (0.2–1.4)	
Tri/quadriplegia	0.7 (0.6–0.8) ^1^	0.8 (0.6–1.2)	0.6 (0.3–1.1)		0.6 (0.2–2.1)		0.4 (0.2–0.9) ^2^	
GMFCS Levels								
I–II	Ref							
III–V	0.7 (0.6–0.8) ^1^	0.7 (0.5–0.9) ^2^	0.6 (0.3–1.1)		0.4 (0.1–1.3)		0.7 (0.4–1.3)	
MACS Levels ^3^								
I–II	Ref							
III–V	0.8 (0.6–0.9) ^2^	0.8 (0.6–1.2)	0.5 (0.3–1.0) ^2^	0.4 (0.1–1.0)	0.7 (0.3–1.6)		n/a ^6^	n/a ^6^
Type of Associated Impairments								
Epilepsy ^4^	0.9 (0.8–1.1)		1.1 (0.6–2.2)		0.5 (0.2–1.6)		0.3 (0.1–0.9) ^2^	0.5 (0.1–1.7)
Intellectual ^4^	1.2 (1.0–1.5) ^2^	1.2 (0.9–1.6)	2.9 (1.4–6.3) ^2^	4.7 (1.6–14.3) ^2^	0.5 (0.2–1.7)		0.6 (0.2–1.6)	
Visual ^4^	1.3 (1.1–1.6) ^2^	0.9 (0.6–1.4)	1.2 (0.5–3.3)		0.6 (0.2–1.6)		0.8 (0.3–2.4)	
Hearing ^4^	2.0 (1.6–2.4) ^1^	2.3 (1.5–3.5) ^1^	1.1 (0.5–2.2)		0.3 (0.1–0.8) ^2^	0.3 (0.1–0.8) ^2^	0.8 (0.4–1.4)	
Speech ^4^	1.0 (0.8–1.2)		1.0 (0.5–2.1)		0.4 (0.2–1.1)		0.4 (0.2–0.9) ^2^	0.6 (0.2–1.7)
Number of Associated Impairments								
None	Ref							
1–2	0.8 (0.7–1.0)		0.6 (0.2–1.6)		0.4 (0.9–1.6)		2.0 (0.4–10.7)	
3–5	1.3 (1.0–1.7)		1.5 (0.5–4.5)		0.5 (0.1–2.6)		0.8 (0.2–4.6)	

^1^*p* < 0.001; ^2^ *p* < 0.05; ^3^ MACS data for Ghana was not available; ^4^ Reference category: No impairment; ^5^ All variables found significant in the unadjusted analysis for each country were included in the adjusted model to identify the potential predictors of not receiving rehabilitation services among children with CP in the BCPR; ^6^ Coefficients could not be computed because of lack of data in one or both categories.

## Data Availability

The data presented in this study are available on request from the corresponding author. The research data contain potentially sensitive and identifying patient information and therefore, the data are not publicly available.
